# Is the Energy Cost of Rowing a Determinant Factor of Performance in Elite Oarsmen?

**DOI:** 10.3389/fphys.2022.827932

**Published:** 2022-03-30

**Authors:** Léo Blervaque, Maximilien Bowen, Benjamin Chatel, Emilio Corbex, Etienne Dalmais, Laurent A. Messonnier

**Affiliations:** Laboratoire Interuniversitaire de Biologie de la Motricité EA 7424, Université Savoie Mont Blanc, Chambéry, France

**Keywords:** rowing, energy cost, ergometer rowing, fat oxidation, elite performance

## Abstract

In elite oarsmen, the rowing ergometer is a valuable tool for both training and studying rowing performance determinants. However, the energy cost of rowing, often reported as a determinant of performance, has never been described for ergometer rowing. Therefore, this study aimed to characterize the energy cost of ergometer rowing (ECR) in elite oarsmen, its contribution to 2,000 m performance, and its determinants. This study was conducted on 21 elite oarsmen from the French national team. It included an incremental exercise test up to exhaustion and an all-out performance test over 2,000 m, both conducted on a rowing ergometer. Gas exchange analysis was performed to calculate oxygen uptake and substrate utilization rate. Whole blood lactate concentrations during the incremental test were obtained from the earlobe. During the incremental test, ECR displayed a significant linear increase up to a plateau that reached a mean rowing speed of 5.23 ± 0.02 m⋅s^–1^. The ECR values at 300, 350, and 400 W were positively correlated with performance expressed as the time required to perform the 2,000 m distance on the rowing ergometer. The same ECR values were found to be significantly related to fat oxidation (expressed in percentage of total energy supply) and blood lactate concentrations. This study provides the first description of ECR and of its relationship to exercise intensity on the rowing ergometer in elite oarsmen. ECR appeared to be a factor of performance and interestingly was related to energy supply from fat and blood lactate concentrations.

## Introduction

The official Olympic distance of rowing races is 2,000 m, lasting between 5:45 and 7:20 (min:s) depending on the boat and the crew. The determinants of performance for this type of event are numerous (Ingham et al., 2002), but the most simplified model, meaning the model with the lowest number of parameters, for predicting performance (expressed in m⋅s^–1^), relies on the ratio between power production (J⋅s^–1^) and the energetic cost of the task (J⋅m^–1^) (di Prampero et al., 1971).

In the case of rowing, power production, the numerator in the abovementioned ratio, has been extensively studied and has been a focus for improvement *via* training. Power production depends mainly on energetic and physiological factors (Hagerman et al., 1978; Secher, 1993; Steinacker and Secher, 1993; Maetsu et al., 2005; Izquierdo-Gabarren et al., 2010)3). In that regard, elevated maximal oxygen uptake ($\dot{\text{V}}{\text{O}}_{2max}$, up to 6.9 L⋅min^–1^) (Nielsen and Christensen, 2020), power output associated with $\dot{\text{V}}{\text{O}}_{2max}$ (${\dot{\text{W}}}_{\mathrm{VO2m}\text{ax}}$, ∼550 W), and blood lactate concentrations (∼16 mmol⋅L^–1^) have been reported in rowers during or in response to performance events (Secher, 1993; Nielsen et al., 2002; Nielsen and Christensen, 2020)). Consequently, an elevated maximal oxygen uptake seems to be a prerequisite for successful rowing (Secher, 1993). Similarly, the later the blood lactate accumulation occurs during an incremental rowing test (∼9–15 mmol⋅L^–1^ posttest), the better the performance (Messonnier et al., 1997). It is also noteworthy that open-class elite rowers are characterized by large body dimensions (height > 190 cm and body weight > 90 kg) (Cosgrove et al., 1999; Ingham et al., 2002).

On the contrary, to the best of our knowledge, the importance of the energy cost of rowing, the denominator in the performance predicting ratio, for 2,000 m performance has rarely been considered, neither in recreational nor in elite rowers. Previously, the energy cost of rowing has been described for on-water rowing (two-oared racing shell with coxswain) (di Prampero et al., 1971). In this study, the energy cost was modeled from the mechanical power required to maintain a given speed against estimated resistance. The absence of oxygen uptake measurement due to inherent constraints of environmental conditions prevented the authors from considering the cost of rowing as a determinant of performance.

Extensively used for training, rowing ergometers are also valuable tools for athlete testing (Lamb, 1989). In fact, rowing ergometer exercise accurately simulates the metabolic demand of on-water rowing and allows rigorous measurement of physiological and metabolic parameters (Lamb, 1989; Rossi et al., 2015; Bourdin et al., 2017). For this reason, elite athletes carry out performance tests, as well as ${\dot{\text{V}}}_{\mathrm{O2max}}$ and lactate threshold determination, using the rowing ergometer. These assessments constitute important parts of the selection process and the annual evaluation routine for elite rowers (Maetsu et al., 2005). Another advantage of this device is that it can provide other useful evaluation parameters, including pace. Thus, interest arose to assess the energy cost of ergometer rowing (ECR), its changes with pace, and its links with performance. Furthermore, the importance of the metabolic source of energy (carbohydrates and fat) on the energy cost of rowing has not been paid attention so far. However, such knowledge is of great importance for the choice of training modalities, insofar as these modalities determine specific improvements for each of the metabolic pathways involved (Gibala et al., 2006). For the French rowing team, the training program is separated into three intensity zones (i.e., moderate for zone 1, heavy for zone 2, and severe for zone 3) separated by the lactate thresholds 1 and 2 (Beneke et al., 2003; Messonnier et al., 2005a). A high training volume is performed at the upper limit of the first zone (Messonnier et al., 2005a), which is systematically higher (∼75%${\dot{\text{W}}}_{\mathrm{VO2max}})$ than exercise-intensity corresponding to the maximal fat oxidation rate (Fat_max_; ∼45–55%${\dot{\text{W}}}_{\mathrm{VO2max}})$ (Brooks and Trimmer, 1996; Rømer et al., 2020). The neglect of low-intensity training is questionable as fat oxidation capacities could be important even for performance during high-intensity exercise (Messonnier et al., 2005b) and metabolic flexibility (San-Millán and Brooks, 2018).

The aim of this study was to (i) describe theECR in French elite rowers, (ii) better understand ECR determinants, and (iii) explore ECR contribution to 2,000 m performance. Specifically, we hypothesized that the ECR and its determinants can play a significant role in ergometer rowing performance.

## Materials and Methods

### Participants

Twenty-one heavyweight male rowers of international level, including two Olympic gold medalists, participated in this study. Data were collected during the annual testing procedure of the athlete. Data obtained between December 2017 and December 2019 were included. This study has been approved by the local ethics (CERUSMB, n° EOFPA-2017) committee and was performed in accordance with the Declaration of Helsinki.

### Devices

A wind-resistance braked rowing ergometer (Concept II model D, fixed, Morrisville, VT, USA) was used for all the tests. Power and heart rate were continuously recorded during the tests. For analysis of expired gases, the subjects breathed through a two-way mouthpiece (Hans Rudolph 2700, Kansas City, MO) connected to a low-resistance, low-dead space mixing chamber (∼2 L). Expired gas fractions were analyzed with O_2_ and CO_2_ analyzers (D-Fend Datex, Helsinki, Finland and S3A/I Ametek, Pittsburgh, PA, respectively). During the time of analysis, expired gases were collected in a Tissot spirometer. More details are provided in the Supplementary Material.

### Incremental Exercise Up to Exhaustion

This test was conducted to obtain maximal oxidative capacities of the athletes as well as to characterize the evolution of parameters with the increase of exercise intensity. The incremental test started at 200 W, and the increment between two successive steps was 50 W. Each step consisted of 3 min rowing and 0.4–0.5 min of rest to complete a blood sample at the earlobe (*vide infra*). The stroke rate was free. The procedure was performed up to exhaustion. During the test, the expired gases were sampled during the last 30 s of each step, analyzed for gas fractions of O_2_ and CO_2_ using gas analyzers (see *Devices*), and collected using the Tissot spirometer (Messonnier et al., 1997). The measurement obtained were used for the calculation of oxygen uptake ($\dot{\text{V}}{\text{O}}_{2}$, in L⋅min^–1^), CO_2_ production ($\dot{\text{V}}{\text{CO}}_{2}$, in L⋅min^–1^), and respiratory exchange ratio (RER = $\dot{\text{V}}{\text{CO}}_{2}/\dot{\text{V}}{\text{O}}_{2}$). More details about gas analysis, calculations, and criterions used for $\dot{\text{V}}{\text{O}}_{2\text{max}}$ achievement are provided in the Supplementary Material. At the end of each step, the blood lactate concentration was analyzed (lactate analyzer 2300 STAT Plus™, YSI, Ohio, USA) from a 20 μl capillary whole blood sample from the hyperemic earlobe, as previously described (Geyssant et al., 1985). The values of mechanical and cardioventilatory parameters obtained at 2 and 4 mmol⋅L^–1^ of lactate concentration were extrapolated from the lactate vs. *ad hoc* parameter relationships using a polynomic fitting.

### Performance Test

The rowers performed a simulated 2,000 m distance as fast as possible on the rowing ergometer. This test was included in the selection process of athletes for the French national team. The time required to cover the distance and the associated speed were used as performance criteria.

### Calculation

The distance ran through each step of the incremental test was calculated as follows:


(1) $$D\left(m\right)=\frac{steptime\left(s\right)}{pace\left(s\cdot m^{-1}\right)}$$

where, the pace is derived from the power according to the following formula (provided by the manufacturer):


(2) $$Pace\left(s\text{⋅}m^{-1}\right)=3\sqrt{\frac{2.8}{Power\left(W\right)}}$$

The mean speed for each step was then computed from distance and exercise time. The energy cost of rowing for each step was calculated from ${\dot{\text{V}}}_{\mathrm{O2}}$(ml⋅min^–1^), blood lactate accumulation between two successive measurements (Δ[La^–^]_b_, mmol⋅L^–1^), body mass (kg), and mean step speed (m⋅min^–1^). To take into account the growing contribution of the non-oxidative glycolytic pathway in the energy supply with increasing exercise intensity, a metabolic equivalent of lactate of 3.3 mlO_2_⋅kg^–1^⋅mmol⋅L^–1^ of Δ[La^–^]_b_^–1^ was used (Margaria et al., 1963). Therefore, ECR can be assessed as follows:


(3) $$ECR\left(mlO_{2}\text{⋅}m^{-1}\right)=\frac{\dot{V}O_{2}+\left(\frac{d{\left[La-\right]}_{b}}{dt}\times 3.3\times bodymass\right)}{meanstepspeed}$$

The oxidation rates of fat and carbohydrates (CHO) (g⋅min^–1^) were indirectly estimated from ${\dot{\text{V}}}_{\mathrm{O2}}$ (L⋅min^–1^) and ${\dot{\text{V}}}_{\mathrm{CO2}}$ (L⋅min^–1^) according to the equation proposed by Péronnet and Massicotte (1991). The oxidation of protein was considered negligible. The conversion from g to kcal was made according to energetic equivalents for fat and CHO (Jeukendrup and Wallis, 2005). The detailed equations are provided below:


(4) $$Fatoxidation\left(kcal/min\right)=\left(\dot{V}O_{2}\times 1.695-\dot{V}CO_{2}\times 1.701\right)\times 9.75$$

(5) $$CHOoxidation\left(kcal/min\right)=\left(\dot{V}CO_{2}\times 4.585-\dot{V}O_{2}\times 3.226\right)\times 4.07$$

(6) $$Fatoxidation\left(\%\right)=\frac{Fatoxidation}{Fatoxidation+CHOoxidation}$$

### Data Analysis

Data are presented as mean with SD. Normality was graphically tested using the quantile-quantile plot method (Aldor-Noiman et al., 2013). The normality of the residual distribution was tested for each model. Correlation coefficients were obtained using the Pearson method (Good, 2009). The evolution of ECR data with increasing speed was analyzed using a linear mixed effect (LME) model with the “Speed” factor as the fixed effect and the “Subject” factor as the random effect. The *post-hoc* multiple comparisons were corrected using the false discovery rate method (Benjamini, 2010). All data were analyzed using RStudio software (RStudio Team, 2021), and the LME was fitted using the R package *nmle* (Pinheiro et al., 2014).

## Results

Demographic, anthropometric, and physiological characteristics of the rowers along with their performance data are reported in Table 1. The extrapolated values of $\dot{\text{V}}{\text{O}}_{2}$ and power output at 4 mmol⋅L^–1^ of [La^–^]_b_ (V.O2BLC4 and ${\dot{\text{W}}}_{\mathrm{BLC4}}$, respectively; Table 1) corresponded to 95% ± 4% and 89% ± 8% of their corresponding maximal counterparts ($\dot{\text{V}}{\text{O}}_{2max}$ and ${\dot{\text{W}}}_{\mathrm{VO2max}},$respectively; Table 1).

**TABLE 1:** Demographic, anthropometric, physiological, and performance characteristics of the rowers (*n* = 21).

Demography/Anthropometry	
Age (years)	25.3 (3.7)
Height (m)	1.91 (0.05)
Weight (kg)	88.3 (5.9)
**Maximal incremental test**	
[La-]_b, max_ (mmol⋅L^–1^)	8.62 (3.19)
ECR_max_ (mLO_2_⋅kg^–1^⋅m^–1^)	0.21 (0.01)
$\dot{\text{V}}{\text{O}}_{2max}$ (L⋅min^–1^)	5.67 (0.36)
$\dot{\text{V}}{\text{O}}_{2max}$ (mL⋅min^–1^⋅kg^–1^)	64.6 (3.3)
$\dot{\text{V}}{\text{O}}_{2\mathrm{BLC2}}$ (L⋅min^–1^)	4.80 (0.43)
$\dot{\text{V}}{\text{O}}_{2\mathrm{BLC4}}$ (L⋅min^–1^)	5.37 (0.36)
${\dot{\text{W}}}_{\bar{\text{V}}\mathrm{O2max}}\left(W\right)$	437 (43)
$\dot{\text{W}}\mathrm{BLC2}\left(W\right)$	328 (32)
$\dot{\text{W}}\mathrm{BLC4}\left(W\right)$	389 (32)
${\dot{\text{W}}}_{\text{max}}\left(W\right)$	455 (35)
HR_max_ (bpm)	189 (8)
**2,000 m performance rowing ergometer test**	
Time over 2,000 m (min)	6.00 (0.13)
Speed over 2,000 m (m⋅s^–1^)	5.56 (0.12)

*ECR, energy cost of rowing; $\dot{\text{V}}{\text{O}}_{2},$oxygen uptake; [La^–^]_b_, blood lactate concentration; $\dot{\text{W}}$, power; HR, heart rate. Data are presented as mean (SD).*

**FIGURE 1:**
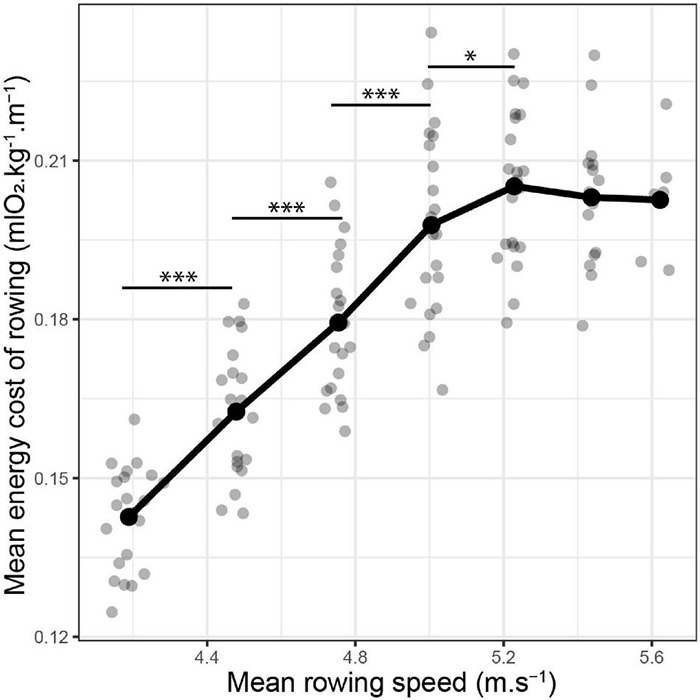
Evolution of the energy cost of rowing (ECR) with rowing speed. Both ECR and speed were recorded for each step of a maximal incremental test on a rowing ergometer. Linear mixed-effect model: speed effect: *p* < 0.001. *Post-hoc*: *p < 0.05; ^***^p < 0.001.

The mean values of ECR (mlO_2_⋅kg^–1^⋅m^–1^) for each step of the incremental test are displayed in Figure 1. Individual ECR kinetics are provided in Supplementary Figure 1. An increase in rowing speed had a significant impact on ECR (LME: speed effect: *p* < 0.001). ECR significantly and linearly increased with the speed until 5.23 ± 0.02 m⋅s^–1^ (Figure 1). From this point, the steepness of the speed vs. ECR relationship decreased drastically, delineating a pseudo-plateau (Figure 1). The maximal value of ECR reached during the maximal incremental test on the rowing ergometer was 0.21 ± 0.01 mlO_2_⋅kg^–1^⋅m^–1^.

Significant correlations between anthropometric parameters (body mass and height) and speed over the 2,000 m were observed (*r* = 0.76; *p* < 0.001 and *r* = 0.56; *p* = 0.01, respectively). As expected, a significant predictive value of performance for the $\dot{\text{V}}{\text{O}}_{2max}$ (*r* = 0.64; *p* < 0.001) and ${\dot{\text{W}}}_{\mathrm{VO2max}}$ (*r* = 0.60; *p* < 0.001) was also found.

Table 2 reports correlations between ECR measured at the different steps and performance. Interestingly, ECR values obtained below the 400 W step, corresponding to 5.23 ± 0.02 m⋅s^–1^ of speed, were negatively and significantly correlated to performance. On the contrary, ECR during the plateau phase (above 5.23 ± 0.02 m⋅s^–1^) no longer correlated with performance.

**TABLE 2:** Correlation coefficients and probabilities between ECR (calculated at the different steps of the incremental exercise, mlO_2_⋅kg^–1^⋅m^–1^), and performance over a 2,000 m rowing ergometer trial (assessed by mean speed, m⋅s^–1^).

Intensity (W)	*R*	*P*-value
200	−0.47	0.03
250	−0.60	<0.001
300	−0.66	<0.001
350	−0.65	<0.001
400	−0.65	<0.001
450	−0.36	0.15
500	−0.06	0.91

**FIGURE 2:**
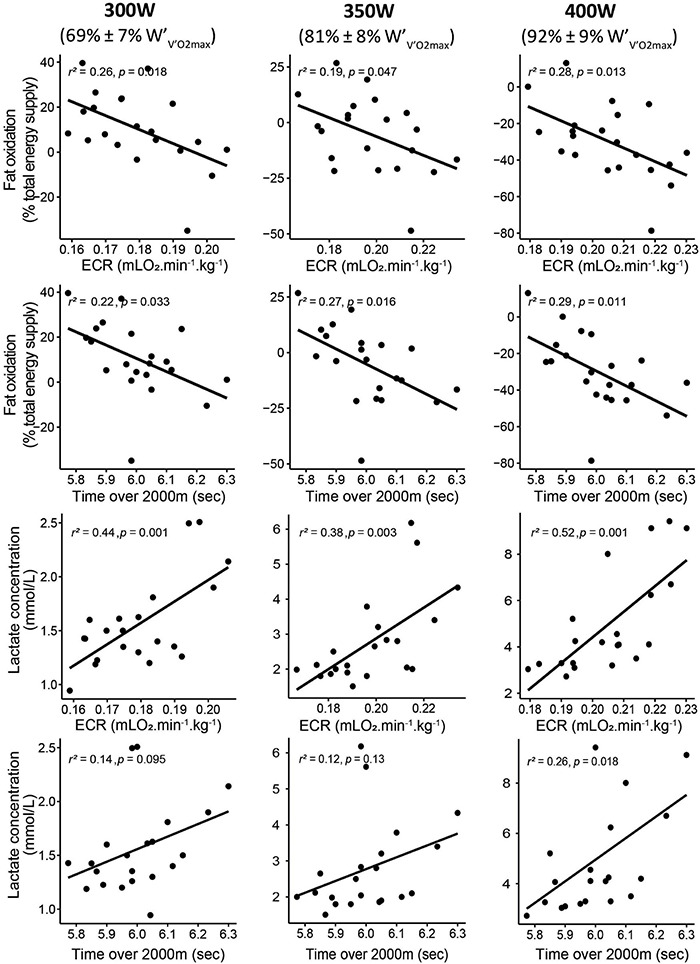
Correlations between fat oxidation, lactate accumulation, the ECR, and performance over 2,000 m rowing. For each parameter, the correlations were displayed for an intensity corresponding to 300 W (left panel), 350 W (middle panel), and 400 W (right panel). $\dot{\text{W}}$, power; $\dot{\text{V}}$O_2_, oxygen uptake.

The proportion of energy provided by fat oxidation was negatively correlated with ECR for exercise intensity of 300 W (mainly below ${\dot{\text{W}}}_{\mathrm{BLC2}}$), 350 W (mainly between ${\dot{\text{W}}}_{\mathrm{BLC2}}$ and ${\dot{\text{W}}}_{\mathrm{BLC4}}$), and 400 W (mainly above ${\dot{\text{W}}}_{\mathrm{BLC4}}$) (Figure 2). Fat oxidation at steps corresponding to 300, 350, and 400 W was also negatively correlated with the time necessary to perform 2,000 m distance (Figure 2). At the same steps, the RER was positively correlated with the time necessary to perform 2,000 m distance (Supplementary Figure 2). [La^–^]_b_ at 300, 350, and 400 W was positively correlated with ECR (Figure 2) and negatively correlated with fat oxidation (Supplementary Figure 3).

## Discussion

This study is the first describing (i) the metabolic ECR as a function of speed during ergometer rowing in elite oarsmen, (ii) the relationship between ECR and ergometer rowing performance, and (iii) the contribution of fat oxidation and lactate accumulation in ECR. The main findings are as follows: (i) ECR increased with rowing speed until reaching a plateau (at 5.23 ± 0.02 m⋅s^–1^ in this study), (ii) ECR at each rowing intensity/velocity before the plateau was strongly correlated with rowing performance (2,000 m time trial), and (iii) ECR during this submaximal phase is negatively and positively related to fat oxidation and blood lactate accumulation, respectively.

To our knowledge, the energy cost of rowing has been described only once. This description has been made on-water (di Prampero et al., 1971). Due to this real-life experimental design, oxygen uptake was not measured, and the energy cost of rowing was calculated from the estimated mechanical power at a given speed. A few decades ago, the rowing ergometer emerged as a major tool for on-land training. At present, it constitutes the foremost instrument to evaluate the physical ability and performance of oarsmen and oarswomen. The calculator of the ergometers displays a lot of interesting information such as the stroke rate, the power output, and the (artificial) speed. The stationary nature of the ergometer also allows a myriad of possible physiological measures including oxygen uptake and blood lactate concentration. In the aggregate, these factors make it possible to calculate ECR on the rowing ergometer. Although different by nature, it is nevertheless interesting to point out similarities between the on-water ECR (di Prampero et al., 1971) and the present on-land ECR. For intensities below 5.2 m⋅s^–1^, the energy cost of rowing described in this study was of the same magnitude order and followed the same increase in function of speed as the one predicted by the equation of di Prampero (1971) (Figure 1). The absence of data equal or superior to 5.2 m⋅s^–1^ during the on-water study (di Prampero et al., 1971) did not allow us to conclude whether the plateau of ECR reached in our study (Figure 1) is specific to ergometer rowing or can be also found in on-water rowing.

In this study, we intended to consider the non-oxidative glycolytic contribution for ECR calculation because (i) part of the lactate produced during exercise is reused as a substrate mainly by oxidation (Brooks and Gaesser, 1980; Miller et al., 2002; Emhoff et al., 2013) and (ii) lactate oxidation is taken into account by the $\dot{\text{V}}{\text{O}}_{2}$, a lactate O_2_ equivalent (Margaria et al., 1963) was applied to the net lactate accumulation (Δ[La^–^]_b_^–1^), which therefore constitutes only a fraction of the total lactate produced (Maciejewski et al., 2013). Despite the fact that this non-oxidative glycolytic contribution for ECR calculation was taken into account, ECR plateaued at high exercise intensity. A likely hypothesis for the onset of the plateau could be an underestimation of the non-oxidative glycolytic contribution at this intensity. In fact, the inertia of lactate transport from the active skeletal muscles to the blood, which can take several minutes (Freund and Zouloumian, 1981), results in an underestimation of blood lactate accumulation and consequently of ECR. To keep the linearity of the rowing speed vs. ECR relationship, a [La^–^]_b_ of 12.2 ± 5.7 mmol⋅L^–1^ instead of 7.9 ± 3.0 mmol⋅L^–1^ should be reached at 5.4 m⋅s^–1^. While these values are realistic, further studies would be necessary to test this hypothesis. At present, the underlying mechanism explaining the appearance of this ECR plateau remains unclear.

The athletes involved in this study were part of the elite French rowing team. The performances obtained over 2,000 m (Table 1) were close to the best performance described in the literature (Boegman et al., 2020). As expected, $\dot{\text{V}}{\text{O}}_{2max}$ or ${\dot{\text{W}}}_{\mathrm{VO2max}}$ were strong determinants of performance (Cosgrove et al., 1999; Ingham et al., 2002). The strong correlation found between performance and body mass drove us to express the cost of rowing as a function of body mass.

In this context, this study is the first to report that the energy cost of rowing (mlO_2_⋅kg^–1^⋅m^–1^) was strongly correlated with rowing performance over 2,000 m (Table 2). This original result echoes the previous report of Cosgrove et al. (1999), showing a negative and significant correlation between the $\dot{\text{V}}{\text{O}}_{2}$ at 4 m⋅s^–1^ and the rowing performance over 2,000 m in club rowers. The energy cost of rowing is mainly determined by the speed and the associated metabolic demand and also by (i) the contribution of the different metabolic pathways and (ii) both metabolic and mechanical efficiencies (Anton-Kuchly et al., 1984; Coyle et al., 1992; Rosenbaum et al., 2003). The latter two indicate that muscle characteristics (e.g., fiber type distribution, surface, and enzyme activities) and technical skills may play important roles in the ECR. During an incremental rowing test, the stroke rate progressively increases. The effect of this stroke rate on efficiency has previously been investigated (Hofmijster et al., 2009; Lindenthaler et al., 2018). The increase of stroke rate theoretically raises the metabolic rate needed to move the mass of the rower during the recovery phase (Lindenthaler et al., 2018). Surprisingly, while this should lead to a decrease in gross efficiency, previous studies reported an increase in efficiency when the work rate and, concomitantly, the stroke rate increase (Lindenthaler et al., 2018). This means that the slope of the increase in ECR observed in this study as a function of work rate should have been even steeper if rowing efficiency had not increased concomitantly.

Interestingly, we observed that the contribution of fat oxidation to energy supply for moderate-intensity exercises (i.e., 300 W, mainly below ${\dot{\text{W}}}_{\mathrm{BLC2}}$) correlated with ECR (Figure 2). This correlation suggests that the rowers who had the lower ECR were also those who drove a larger part of the energy supply from fat oxidation. This negative relationship between ECR and fat oxidation is somewhat surprising since the number of ATP produced/oxygen consumption ratio is lower for fat than for carbohydrates. Previous reports showed that specific blockade of type I fibers during exercise increases $\dot{\text{V}}{\text{O}}_{2}$ for a given exercise intensity and thus the energy cost of the task (Krustrup et al., 2008). Therefore, this fiber type-specific efficiency can be a clue in the understanding of our results. Since a greater fat oxidation rate occurs in slow-twitch fibers (Lowry et al., 1978), the higher fat oxidation observed for the rowers who displayed the lower ECR may reflect higher recruitment and/or proportion of slow-twitch fibers in those rowers. However, this last point remains speculative, and its further confirmation is essential.

Surprisingly, similar correlations between ECR and fat oxidation have been observed for high exercise intensities, typically between ${\dot{\text{W}}}_{\mathrm{BLC2}}$ and ${\dot{\text{W}}}_{\mathrm{BLC4}}$ and above ${\dot{\text{W}}}_{\mathrm{BLC4}}$(Figure 2). These correlations where fat oxidation is virtually absent (RER > 1.00) seem aberrant at first sight and require us to interpret them with caution. However, the present results are reminiscent of a previous study, showing that the decrease in RER in the severe-intensity domain (i.e., above ${\dot{\text{W}}}_{\mathrm{BLC4}}$; Beneke et al., 2003), induced by endurance training, was correlated with the concomitant increase in β-HAD activity, an enzyme involved in fat oxidation (Messonnier et al., 2005b). One possible explanation for these correlations in the severe-intensity domain is to consider the heterogeneity of the active skeletal muscles. In that context and considering that RER is the integration of O_2_ consumption and CO_2_ production of each muscle fiber involved in the exercise, a mean RER > 1.00 may not mean that all muscle fibers display a CO_2_ production superior to O_2_ consumption. Thus, for an RER of 1.00, some very oxidative fibers may work at RER < 1.00, while more glycolytic fibers may exercise at RER > 1.00. Thus, we can put forward the hypothesis that the difference between two RER > 1.00, e.g., 1.05 vs. 1.15 can be the result of a greater extent of fiber displaying individual RER < 1.00 in the first case, meaning that some fibers may still utilize fat. Type I fiber is the fiber type most likely to maintain an RER < 1.00 during high-intensity exercise, due to their ability of fat oxidation (high β-HAD activity in this fiber type) (Nicol and Johnston, 1981). Consistent with this hypothesis, previous studies have shown that the high energy cost of a task has been related to early-type II fiber recruitment (Krustrup et al., 2008). Completion of 2,000 m in ergometer rowing takes approximately 6 min and is performed close to ${\dot{W}}_{\mathrm{VO2max}}$. It has been shown that improvement in time to exhaustion at ${\dot{\text{W}}}_{\mathrm{VO2max}}$ (lasting 5–8 min) was linked to improvement in β-HAD activity and decreased RER (Messonnier et al., 2005b). These latest results would support the correlation between performance over 2,000 m and both RER and fat oxidation found in this study.

However, considering the correlations between fat oxidation and ECR or performance as a direct (cause-effect) relationship while fat oxidation is minimal or absent (Figure 2 and Supplementary Figure 2) is highly questionable. In that context, the correlations we observed between lactate concentrations and fat oxidation (Supplementary Figure 3) and ECR (Figure 2) allow for other interpretations. Lactate accumulation can directly inhibit fat oxidation (San-Millán and Brooks, 2018) *via* at least two mechanisms. Lactate accumulation (1) inhibits lipolysis (Boyd et al., 1974; Liu et al., 2009) and (2) induces the formation of malonyl-CoA, which inhibits the carnitine-palmitoyltransferase-1 (CPT1) and consequently the entry of free fatty acids into the mitochondria (McGarry et al., 1977). From this point of view, the apparent role of improved fat oxidation and decreased blood lactate accumulation on ECR and rowing performance may reflect the better mitochondrial function of the best rowers (San-Millán and Brooks, 2018). This latter interpretation is also in accordance with the previously evoked higher recruitment and/or proportion of slow-twitch fibers in best rowers since these fibers are known to use fat and lactate as energy substrates. As a whole, this latter interpretation would indicate that ECR is related to the metabolic flexibility of the rowers (San-Millán and Brooks, 2018). Thus, one possible interpretation of our results could be that the better the rowers’ metabolic flexibility, the lower their ECR and the higher their performance. However, this statement must be taken with caution given the correlational nature of this study.

The present results may also have important implications for the training of high-level rowers. Our results suggest that the improvement of fat oxidation may be a training goal. In that sense, the role of exercise training at moderate intensity (below ${\dot{\text{W}}}_{\mathrm{BLC2}}$), known to enhance fat oxidation abilities (Schrauwen et al., 2002), is essential. This type of training may contribute to improving rowing performance.

Finally, the question of the transferability of these results to on-water 2,000 m races is of interest. The reliability of the rowing ergometer in the simulation of on-water physiological and mechanical demands has been demonstrated earlier (Hagerman, 1984; Lamb, 1989; Vogler et al., 2010). While the ECR has never been formally assessed by the on-water incremental test, the changes of ECR as a function of speed match the one predicted for on-water rowing by di Prampero et al. (1971), which supports the transferability of our results. However, the complex nature of on-water rowing, in particular, the technical and environmental determinants makes it essential to carry out additional studies to ensure this transferability.

### Limitations

The main limitation of this study is the nature of the task used to determine the ECR. In fact, the energy cost of the task on the rowing ergometer is ruled by less determinants (technical skills, environmental constraints, etc.) than on-water rowing, which can explain lower ECR value (Vogler et al., 2010). This limitation should be addressed in the future with ECR determination for on-water rowing, using a wearable gas analyzer device for instance. The complex nature of on-water rowing (e.g., weather) can also lead to a reduced performance gap between the lightweight and heavyweight rowers than observed on the rowing ergometer. However, focusing here on a homogenous group of heavyweight rowers and expressing ECR as a function of body mass attenuates this limit. The similarities pointed out above between ECR described for on-water rowing and the one described in this study imply that the results provided in this study could be reproducible during on-water experiments.

## Conclusion

This study is the first to describe the ECR, taking into account both oxidative and glycolytic non-oxidative contribution, during ergometer rowing. ECR followed a linear increase as a function of speed until reaching a plateau. The ECR in intensities below the plateau was found to be a determinant of ergometer rowing performance over 2,000 m in elite oarsmen. This study also provides new insights into the possible contribution of fat oxidation and delayed lactate accumulation to rowing performance.

## Data Availability Statement

The raw data supporting the conclusions of this article will be made available by the authors, without undue reservation.

## Ethics Statement

The studies involving human participants were reviewed and approved by Comité d’Éthique de la Recherche - Université Savoie Mont Blanc (CER – USMB). The patients/participants provided their written informed consent to participate in this study.

## Author Contributions

LB was involved in hypothesis formulation, critical analysis of the data, and wrote the manuscript. MB, BC, EC, and ED were involved in study design, data collection, and analysis. LM was involved in study design and supervision, data collection, supervision of hypothesis formulation, and data analysis. All authors did a critical reading of the manuscript.

## Conflict of Interest

The authors declare that the research was conducted in the absence of any commercial or financial relationships that could be construed as a potential conflict of interest.

## Publisher’s Note

All claims expressed in this article are solely those of the authors and do not necessarily represent those of their affiliated organizations, or those of the publisher, the editors and the reviewers. Any product that may be evaluated in this article, or claim that may be made by its manufacturer, is not guaranteed or endorsed by the publisher.

## References

[B1] Aldor-Noiman S. Brown L. D. Buja A. Rolke W. Stine R. A. (2013). The power to see: a new graphical test of normality. *Am. Stat.* 67 249–260. 10.1080/00031305.2013.847865

[B2] Anton-Kuchly B. Roger P. Varene P. (1984). Determinants of increased energy cost of submaximal exercise in obese subjects. *J. Appl. Physiol. Respir. Environ. Exerc. Physiol.* 56 18–23. 10.1152/jappl.1984.56.1.18 6693318

[B3] Beneke R. Hütler M. Von Duvillard S. P. Sellens M. Leithäuser R. M. (2003). Effect of test interruptions on blood lactate during constant workload testing. *Med. Sci. Sports Exerc.* 35 1626–1630. 10.1249/01.MSS.0000084520.80451.D5 12972887

[B4] Benjamini Y. (2010). Discovering the false discovery rate. *J. R. Stat. Soc. Ser. B Stat. Methodol.* 72 405–416. 10.1111/j.1467-9868.2010.00746.x

[B5] Boegman S. Stellingwerff T. Shaw G. Clarke N. Graham K. Cross R. (2020). The impact of individualizing sodium bicarbonate supplementation strategies on world-class rowing performance. *Front. Nutr.* 7:138. 10.3389/fnut.2020.00138 33015117 PMC7509055

[B6] Bourdin M. Lacour J. R. Imbert C. Messonnier L. A. (2017). Factors of rowing ergometer performance in high-level female rowers. *Int. J. Sports Med.* 38 1023–1028. 10.1055/s-0043-118849 28965342

[B7] Boyd A. E. Giamber S. R. Mager M. Lebovitz H. E. (1974). Lactate inhibition of lipolysis in exercising man. *Metabolism* 23 531–542. 10.1016/0026-0495(74)90081-X 4828442

[B8] Brooks G. A. Gaesser G. A. (1980). End points of lactate and glucose metabolism after exhausting exercise. *J. Appl. Physiol.* 49, 1057–1069. 10.1152/JAPPL.1980.49.6.1057 7440296

[B9] Brooks G. A. Trimmer J. K. (1996). Glucose kinetics during high-intensity exercise and the crossover concept. *J. Appl. Physiol. (Bethesda, Md. 1985)* 80 1073–1075. 10.1152/jappl.1996.80.3.1073 8964726

[B10] Cosgrove M. J. Wilson J. Watt D. Grant S. F. (1999). The relationship between selected physiological variables of rowers and rowing performance as determined by a 2000 m ergometer test. *J. Sports Sci.* 17 845–852. 10.1080/026404199365407 10585164

[B11] Coyle E. F. Sidossis L. S. Horowitz J. F. Beltz J. D. (1992). Cycling efficiency is related to the percentage of Type I muscle fibers. *Med. Sci. Sports Exerc.* 24 782–788. 10.1249/00005768-199207000-00008 1501563

[B12] di Prampero P. E. Cortili G. Celentano F. Cerretelli P. (1971). Physiological aspects of rowing. *J. Appl. Physiol.* 31 853–857. 10.1152/jappl.1971.31.6.853 5123663

[B13] Emhoff C. A. W. Messonnier L. A. Horning M. A. Fattor J. A. Carlson T. J. Brooks G. A. (2013). Gluconeogenesis and hepatic glycogenolysis during exercise at the lactate threshold. *J. Appl. Physiol*. 114, 297–306. 10.1152/japplphysiol.01202.2012 23239870 PMC8846961

[B14] Freund H. Zouloumian P. (1981). Lactate after exercise in man: I. Evolution kinetics in arterial blood. *Eur. J. Appl. Physiol. Occup. Physiol.* 46 121–133. 7194790 10.1007/BF00428865

[B15] Geyssant A. Dormois D. Barthelemy J. C. Lacour J. R. (1985). Lactate determination with the lactate analyser LA 640: a critical study. *Scand. J. Clin. Lab. Invest.* 45 145–149. 10.3109/00365518509160987 4001823

[B16] Gibala M. J. Little J. P. van Essen M. Wilkin G. P. Burgomaster K. A. Safdar A. (2006). Short-term sprint interval versus traditional endurance training: similar initial adaptations in human skeletal muscle and exercise performance. *J. Physiol.* 575 901–911. 10.1113/jphysiol.2006.112094 16825308 PMC1995688

[B17] Good P. (2009). Robustness of Pearson correlation. *Interstat* 15 1–6.

[B18] Hagerman F. C. (1984). Applied physiology of rowing. *Sports Med. Int. J. Appl. Med. Sci. Sport Exerc.* 1 303–326. 10.2165/00007256-198401040-00005 6390606

[B19] Hagerman F. C. Connors M. C. Gault J. A. Hagerman G. R. Polinski W. J. (1978). Energy expenditure during simulated rowing. *J. Appl. Physiol. Respir. Environ. Exerc. Physiol.* 45 87–93. 10.1152/jappl.1978.45.1.87 670038

[B20] Hofmijster M. J. Van Soest A. J. De Koning J. J. (2009). Gross efficiency during rowing is not affected by stroke rate. *Med. Sci. Sports Exerc.* 41 1088–1095. 10.1249/MSS.0B013E3181912272 19346978

[B21] Ingham S. A. Whyte G. P. Jones K. Nevill A. M. (2002). Determinants of 2,000 m rowing ergometer performance in elite rowers. *Eur. J. Appl. Physiol.* 88 243–246. 10.1007/s00421-002-0699-9 12458367

[B22] Izquierdo-Gabarren M. De Txabarri Expósito R. G. De Villarreal E. S. S. Izquierdo M. (2010). Physiological factors to predict on traditional rowing performance. *Eur. J. Appl. Physiol.* 108 83–92. 10.1007/s00421-009-1186-3 19756709

[B23] Jeukendrup A. E. Wallis G. A. (2005). Measurement of substrate oxidation during exercise by means of gas exchange measurements. *Int. J. Sports Med.* 26(Suppl. 1) S28–S37. 10.1055/s-2004-830512 15702454

[B24] Krustrup P. Secher N. H. Relu M. U. Hellsten Y. Söderlund K. Bangsbo J. (2008). Neuromuscular blockade of slow twitch muscle fibres elevates muscle oxygen uptake and energy turnover during submaximal exercise in humans. *J. Physiol.* 586 6037–6048. 10.1113/jphysiol.2008.158162 18955384 PMC2655428

[B25] Lamb D. H. (1989). A kinematic comparison of ergometer and on-water rowing. *Am. J. Sports Med.* 17 367–373. 10.1177/036354658901700310 2729487

[B26] Lindenthaler J. R. Rice A. J. Versey N. G. McKune A. J. Welvaert M. (2018). Differences in physiological responses during rowing and cycle ergometry in elite male rowers. *Front. Physiol.* 9:1010. 10.3389/fphys.2018.01010 30104984 PMC6077239

[B27] Liu C. Wu J. Zhu J. Kuei C. Yu J. Shelton J. (2009). Lactate inhibits lipolysis in fat cells through activation of an orphan G-protein-coupled receptor, GPR81. *J. Biol. Chem.* 284 2811–2822. 10.1074/jbc.M806409200 19047060

[B28] Lowry C. V. Kimmey J. S. Felder S. Chi M. M. Kaiser K. K. Passonneau P. N. (1978). Enzyme patterns in single human muscle fibers. *J. Biol. Chem*. 253, 8269–8277. 10.1016/s0021-9258(17)34391-0 152314

[B29] Maciejewski H. Bourdin M. Lacour J. R. Denis C. Moyen B. Messonnier L. (2013). Lactate accumulation in response to supramaximal exercise in rowers. *Scand. J. Med. Sci. Sports* 23 585–592. 10.1111/j.1600-0838.2011.01423.x 22288604

[B30] Maetsu J. Jurimae J. Jurimae T. (2005). Monitoring of performance and training in rowing. *Sports Med.* 35 597–617. 16026173 10.2165/00007256-200535070-00005

[B31] Margaria R. Cerretelli P. Diprampero P. E. Massari C. Torelli G. (1963). Kinetics and mechanism of oxygen debt contraction in man. *J. Appl. Physiol.* 18 371–377. 10.1152/jappl.1963.18.2.371 13932994

[B32] McGarry J. D. Mannaerts G. P. Foster D. W. (1977). A possible role for malonyl CoA in the regulation of hepatic fatty acid oxidation and ketogenesis. *J. Clin. Invest.* 60 265–270. 10.1172/JCI108764 874089 PMC372365

[B33] Messonnier L. Aranda-Berthouze S. E. Bourdin M. Bredel Y. Lacour J. R. (2005a). Rowing performance and estimated training load. *Int. J. Sports Med.* 26 376–382. 10.1055/s-2004-821051 15895321

[B34] Messonnier L. Denis C. Prieur F. Lacour J. R. (2005b). Are the effects of training on fat metabolism involved in the improvement of performance during high-intensity exercise? *Eur. J. Appl. Physiol.* 94 434–441. 10.1007/s00421-005-1325-4 15843960

[B35] Messonnier L. Freund H. Bourdin M. Belli A. Lacour J. R. (1997). Lactate exchange and removal abilities in rowing performance. *Med. Sci. Sports Exerc.* 29 396–401. 10.1097/00005768-199703000-00016 9139180

[B36] Miller B. F. Fattor J. A. Jacobs K. A. Horning M. A. Navazio F. Lindinger M. I. (2002). Lactate and glucose interactions during rest and exercise in men: effect of exogenous lactate infusion. *J. Physiol*. 544, 963–975. 10.1113/jphysiol.2002.027128 12411539 PMC2290635

[B37] Nicol C. J. M. Johnston I. A. (1981). Energy metabolism of fast- and slow-twitch skeletal muscle in the rat: thyroid hormone induced changes. *J. Comp. Physiol. B* 142 465–472. 10.1007/BF00688977

[B38] Nielsen H. B. Christensen P. M. (2020). Rower with Danish record in maximal oxygen uptake. *Ugeskr. Laeger* 182:V10190610. 32138820

[B39] Nielsen H. B. Bredmose P. P. Strømstad M. Volianitis S. Quistorff B. Secher N. H. (2002). Bicarbonate attenuates arterial desaturation during maximal exercise in humans. *J. Appl. Physiol.* 93 724–731. 10.1152/japplphysiol.00398.2000 12133884

[B40] Péronnet F. Massicotte D. (1991). Table of nonprotein respiratory quotient: an update. *Can. J. Sport Sci. J. Can. Sci. Sport* 16 23–29. 1645211

[B41] Pinheiro J. Bates D. DebRoy S. Sarkar D. R Core Team (2014). *nlme: Linear and Nonlinear Mixed Effects Models. R Package Version 3.1–117.* Available online at: http://Cran.r-project.org/web/packages/nlme/index.html

[B42] Rømer T. Thunestvedt Hansen M. Frandsen J. Larsen S. Dela F. Wulff Helge J. (2020). The relationship between peak fat oxidation and prolonged double-poling endurance exercise performance. *Scand. J. Med. Sci. Sports* 30 2044–2056. 10.1111/sms.13769 32654310

[B43] Rosenbaum M. Vandenborne K. Goldsmith R. Simoneau J. A. Heymsfield S. Joanisse D. R. (2003). Effects of experimental weight perturbation on skeletal muscle work efficiency in human subjects. *Am. J. Physiol. Regul. Integr. Comp. Physiol.* 285 183–192. 10.1152/ajpregu.00474.2002 12609816

[B44] Rossi J. Piponnier E. Vincent L. Samozino P. Messonnier L. (2015). Influence of ergometer design on physiological responses during rowing. *Int. J. Sports Med.* 36 947–951. 10.1055/s-0035-1548810 26212249

[B45] RStudio Team (2021). *Studio: Integrated Development for R.* Boston, MA: RStudio, Inc., PBC.

[B46] San-Millán I. Brooks G. A. (2018). Assessment of metabolic flexibility by means of measuring blood lactate, fat, and carbohydrate oxidation responses to exercise in professional endurance athletes and less-fit individuals. *Sports Med.* 48 467–479. 10.1007/s40279-017-0751-x 28623613

[B47] Schrauwen P. Van Aggel-Leijssen D. P. C. Hul G. Wagenmakers A. J. M. Vidal H. Saris W. H. M. (2002). The effect of a 3-month low-intensity endurance training program on fat oxidation and acetyl-CoA carboxylase-2 expression. *Diabetes* 51 2220–2226. 10.2337/diabetes.51.7.2220 12086953

[B48] Secher N. H. (1993). Physiological and biomechanical aspects of rowing: implications for training. *Sports Med. Int. J. Appl. Med. Sci. Sport Exerc.* 15 24–42. 10.2165/00007256-199315010-00004 8426942

[B49] Steinacker J. M. Secher N. H. (1993). Advances in physiology and biomechanics of rowing. *Int. J. Sports Med.* 14(Suppl. 1) S1–S2. 10.1055/s-2007-1021214 8262698

[B50] Vogler A. J. Rice A. J. Gore C. J. (2010). Physiological responses to ergometer and on-water incremental rowing tests. *Int. J. Sports Physiol. Perform.* 5 342–358. 10.1123/ijspp.5.3.342 20861524

