# Supplementary material for: Is the Energy Cost of Rowing a Determinant Factor of Performance in Elite Oarsmen?
Source: Front Physiol. 2022 Mar 30;13:827932. doi: 10.3389/fphys.2022.827932 (PMC9005883; doi:10.3389/fphys.2022.827932)
Supplement: Supplementary file 1 [file Table_1.DOCX]

# SUPPLEMENTARY MATERIAL

**Is the energy cost of rowing a determinant factor of performance in elite oarsmen?**

# Authorship

Léo Blervaque^1^, Maximilien Bowen^1^, Benjamin Chatel^1^, Emilio Corbex^1^, Etienne Dalmais^1^, Laurent A. Messonnier^1^.

1. Université Savoie Mont Blanc, Laboratoire Interuniversitaire de Biologie de la Motricité EA7424, Chambéry, France.

# SUPPLEMENTAL MATERIALS & METHODS

A wind-resistance braked rowing ergometer (Concept II model D, fixed, Morrisville, VT, USA) was used for all the tests. A homemade LabView-designed interface (LabView, National Instrument, Austin, USA) was used to continuously display and record the power and heart rate provided by the PM5 monitor associated with the concept 2. Heart rate was recorded with a heart rate strap (Polar H10, Polar Electro, Inc., Kempele, Finland) paired to the PM5. For analysis of expired gases, the subjects breathed through a two-way mouthpiece (Hans Rudolph 2700, Kansas City, MO) connected to a low-resistance, low-dead space mixing chamber (~2 liters). Expired gas fractions were analyzed with O_2_ and CO_2_ analyzers (D-Fend Datex, Helsinki, Finland and S3A/I Ametek, Pittsburgh, PA respectively) connected to the LabView recording interface. During the time of analysis, expired gases were collected in a Tissot spirometer.

**Incremental exercise up to exhaustion**

# During the test, the expired gases were sampled during the last 30 s of each step, analyzed for gas fractions of O_2_ and CO_2_ using gas analyzers (see *Devices*), calibrated before each test using a three-point method with precise gas mixtures (100% nitrogen, 5%CO_2_/12%O_2_, 6%CO_2_/20.9%O_2_) (Messonnier et al., 1997). Expired gas collected in the Tissot spirometer were divided by collection time to obtain minute ventilation ${(\dot{V}}_{E})$ at ambient temperature and pressure, saturated (ATPS). $\dot{V}_{E}$ (ATPS) was converted to $\dot{V}_{E}$ (STPD – standard temperature and pressure, dry) for calculation of oxygen uptake (${\dot{V}O}_{2}$, in L.min^-1^), CO_2_ production (${\dot{V}\mathrm{CO}}_{2}$, in L.min^-1^) and respiratory exchange ratio (RER = ${\dot{V}\mathrm{CO}}_{2}/{\dot{V}O}_{2}$). At the end of each step, the blood lactate concentration was analyzed (lactate analyzer 2300 STAT Plus™, YSI, Ohio, USA) from a 20 µl capillary whole blood sample from the hyperemic earlobe, as previously described (Geyssant et al., 1985). ${\dot{V}O}_{2 \max}$ was considered achieved when a ${\dot{V}O}_{2}$ plateau (appearance of data points outside and below the extrapolated 95% CI of the linear $\dot{V}_{O2}$/$\dot{W}$relationship)(Poole et al., 2008) was observed while work rate increased, or in absence of a plateau, if two of the three following criteria were reached: a RER exceeding 1.1 (Messonnier et al., 1997; Lacour et al., 2007, 2009), an end-exercise blood lactate concentration higher than 9 mmol.L^-1^ and attainment of the theoretical maximal heart rate.

REFERENCES

Geyssant, A., Dormois, D., Barthelemy, J. C., and Lacour, J. R. (1985). Lactate determination with the lactate analyser LA 640: a critical study. *Scand. J. Clin. Lab. Invest.* 45, 145–149. doi:10.3109/00365518509160987.

Lacour, J. R., Messonnier, L., and Bourdin, M. (2007). The leveling-off of oxygen uptake is related to blood lactate accumulation. Retrospective study of 94 elite rowers. *Eur. J. Appl. Physiol.* 101, 241–247. doi:10.1007/S00421-007-0487-7.

Lacour, J. R., Messonnier, L., and Bourdin, M. (2009). Physiological correlates of performance. Case study of a world-class rower. *Eur. J. Appl. Physiol.* 106, 407–413. doi:10.1007/S00421-009-1028-3.

Messonnier, Freund, Bourdin, Belli, and Lacour (1997). Lactate exchange and removal abilities in rowing performance. *Med. Sci. Sports Exerc.* 29, 396–401. doi:10.1097/00005768-199703000-00016.

Poole, D. C., Wilkerson, D. P., and Jones, A. M. (2008). Validity of criteria for establishing maximal O 2 uptake during ramp exercise tests. *Eur. J. Appl. Physiol.* 102, 403–410. doi:10.1007/s00421-007-0596-3.

# SUPPLEMENTAL FIGURE LEGEND

**Supplemental Figure 1:** *Individual trajectories of the energy cost of rowing (ECR) with rowing speed.* Both ECR and speed were recorded for each step of a maximal incremental test on rowing ergometer.

**Supplemental Figure 2:** *Correlations between respiratory exchange ratio (RER), energy cost of rowing (ECR) and performance over 2000m rowing.* For each parameter, the correlations were displayed for an intensity corresponding to 300W (left panel), 350W (middle panel) and 400W (right panel). W’: power; V’O_2_: oxygen uptake.

**Supplemental Figure 3:** *Correlations between lactate accumulation and fat oxidation.* For each parameter, the correlations were displayed for an intensity corresponding to 300W (left panel), 350W (middle panel) and 400W (right panel). W’: power; V’O_2_: oxygen uptake.
