# Supplementary figures and images for: Is the Energy Cost of Rowing a Determinant Factor of Performance in Elite Oarsmen?
Source: Front Physiol. 2022 Mar 30;13:827932. doi: 10.3389/fphys.2022.827932 (PMC9005883; doi:10.3389/fphys.2022.827932)

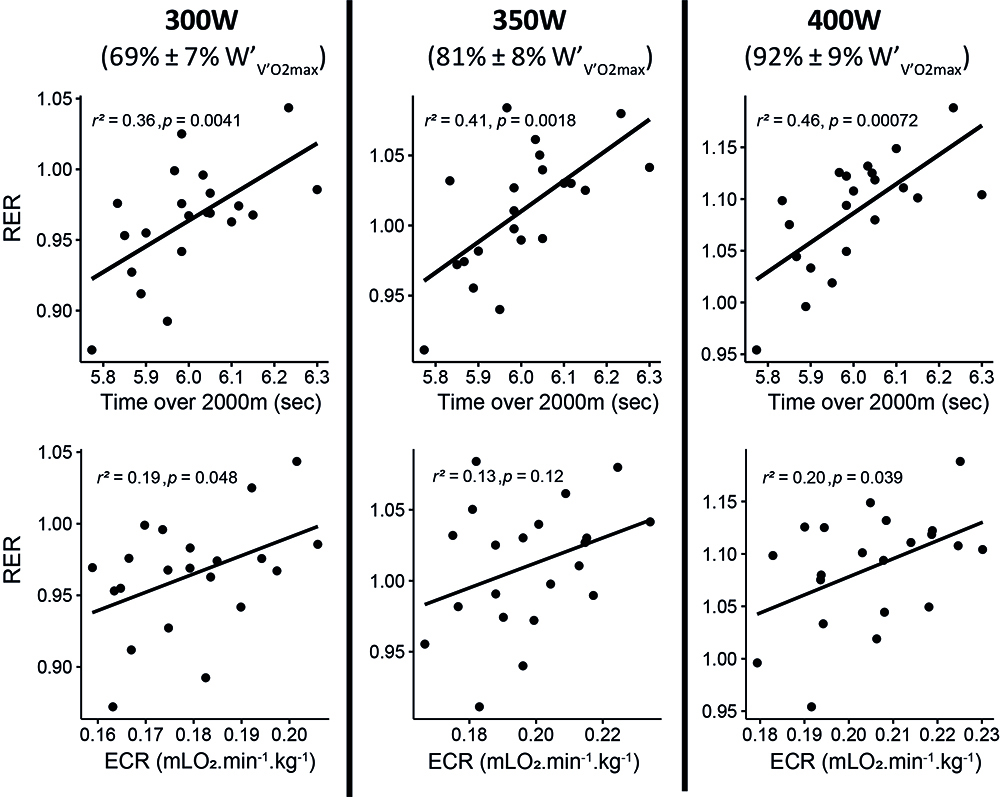

Supplement: Supplementary file 3 [file Image_1.JPEG]

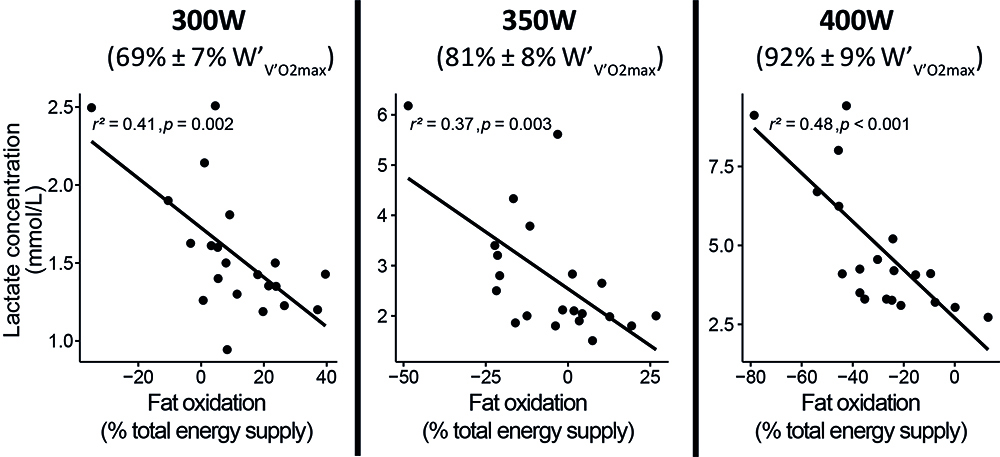

Supplement: Supplementary file 4 [file Image_2.JPEG]

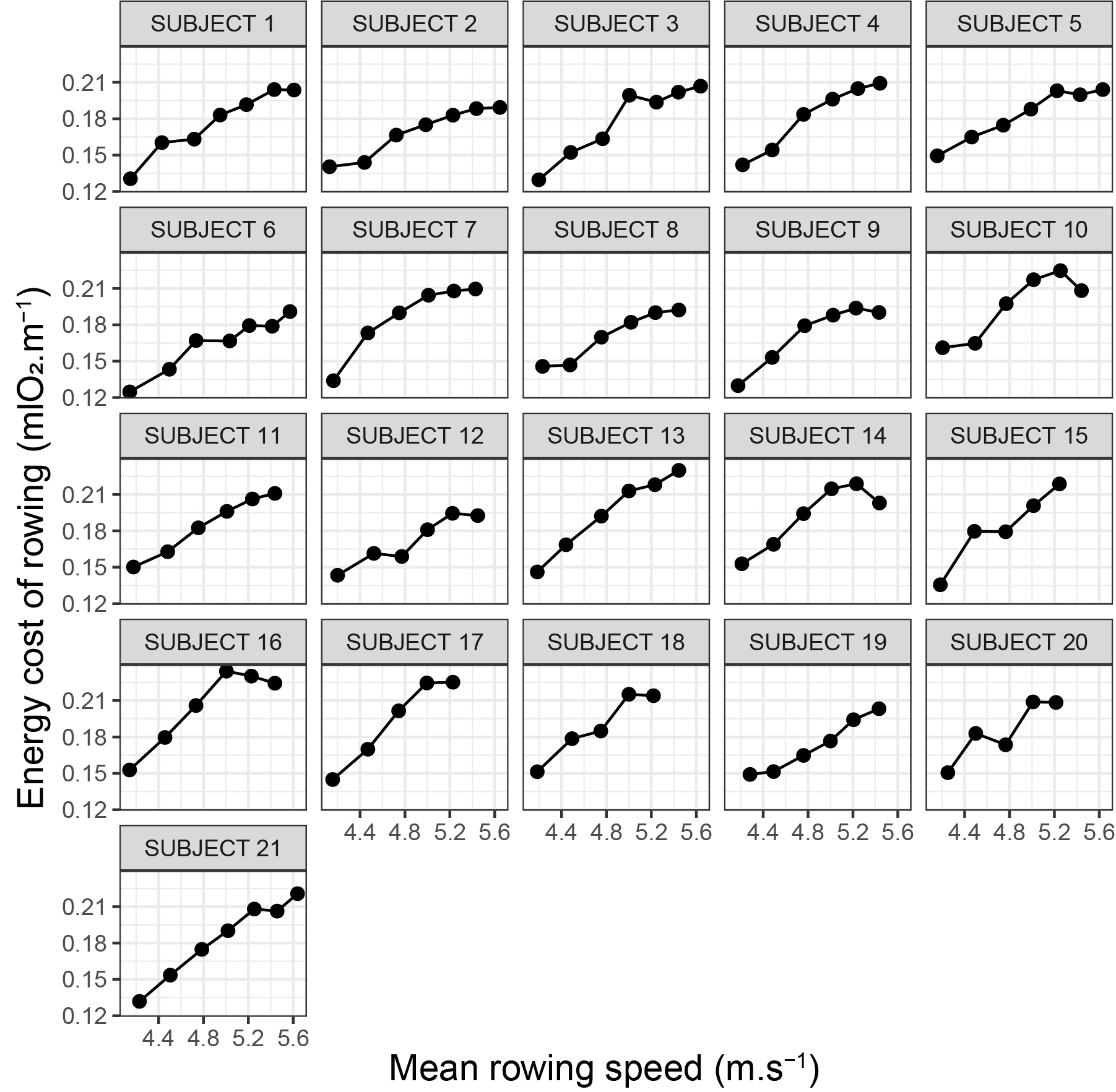

Supplement: Supplementary file 5 [file Image_3.JPEG]
